# Image Harvest: an open-source platform for high-throughput plant image processing and analysis

**DOI:** 10.1093/jxb/erw176

**Published:** 2016-05-03

**Authors:** Avi C. Knecht, Malachy T. Campbell, Adam Caprez, David R. Swanson, Harkamal Walia

**Affiliations:** ^1^University of Nebraska-Lincoln, Holland Computing Center, Lincoln, NE 68583, USA.; ^2^University of Nebraska-Lincoln, Department of Agronomy and Horticulture, Lincoln, NE 68583, USA.

**Keywords:** High throughput computing, image analysis, image processing, large-scale biology, OpenCV, Open Science Grid, open-source software, phenomics.

## Abstract

High-throughput plant phenotyping is an effective approach to bridge the genotype-to-phenotype gap in crops. Phenomics experiments typically result in large-scale image datasets, which are not amenable for processing on desktop computers, thus creating a bottleneck in the image-analysis pipeline. Here, we present an open-source, flexible image-analysis framework, called Image Harvest (IH), for processing images originating from high-throughput plant phenotyping platforms. Image Harvest is developed to perform parallel processing on computing grids and provides an integrated feature for metadata extraction from large-scale file organization. Moreover, the integration of IH with the Open Science Grid provides academic researchers with the computational resources required for processing large image datasets at no cost. Image Harvest also offers functionalities to extract digital traits from images to interpret plant architecture-related characteristics. To demonstrate the applications of these digital traits, a rice (*Oryza sativa*) diversity panel was phenotyped and genome-wide association mapping was performed using digital traits that are used to describe different plant ideotypes. Three major quantitative trait loci were identified on rice chromosomes 4 and 6, which co-localize with quantitative trait loci known to regulate agronomically important traits in rice. Image Harvest is an open-source software for high-throughput image processing that requires a minimal learning curve for plant biologists to analyzephenomics datasets.

## Highlight

Image Harvest is an open-source software for high-throughput image processing and analysis that is integrated with the Open Science Grid and provides computational resources to process large image datasets.

## Introduction

With the advent of next-generation sequencing technologies and development of high-density genotyping platforms for many crop species, the volume of genotypic data being generated has increased exponentially. However, the collection of corresponding phenotypic data has lagged behind and remains a laborious task, often involving destructive measurements. Large-scale phenotypic (phenomics) data for traits of interest are essential to realize the full potential of the enormous progress in crop genomics. The generation of phenomics data for plants is challenging because many of the commercially valuable traits are quantitative, exhibit high variability across diverse environments, and change throughout the plant’s life cycle, thus adding to cost and labor. Further, many phenotypic traits are not amenable for visual scoring and observations on a large scale. These challenges have led to a translational bottleneck, often referred to as the genotype–phenotype gap. This challenge is discussed in several recent reviews (Furbank and Tester, 2011; Sozzani and Benfey, 2011; Gjuvsland *et al.*, 2013; Yang *et al.*, 2013; Brown *et al.*, 2014; Fraas and Luthen, 2015).

To bridge this gap, several efforts in the public sector and industry have started to materialize through the establishment of research centers focusing on the development and applications of high-throughput phenomics technologies (Yang *et al.*, 2013). Image-based plant phenomics is one area becoming increasingly accessible in the public domain [e.g. Australian Plant Phenomics Facility; European Plant Phenotyping Network; PHENOME: The French Plant Phenomics Network (FPPN); High-Throughput Rice Phenotyping Facility (HRPF; Huazhong University, China); Donald Danforth Plant Science Center; University of Nebraska-Lincoln Innovation Campus; LeasyScan at ICRISAT]. These high-throughput imaging platforms allow for non-destructive measurements to be recorded accurately and frequently during the course of an experiment. Such systems utilize a series of cameras to estimate plant growth, temperature, water content, or chlorophyll characteristics on a large scale, and provide researchers with valuable insights into dynamic physiological changes occurring during the course of plant development or in response to environmental changes. These platforms are often fully automated and therefore have lower technical errors compared to traditional phenotyping methods. Overall, the optics-based systems greatly enhance the ability to capture quantitative traits on a temporal and spatial scale, and through the integration of mechanical automation, make high-throughput plant phenotyping accessible to public researchers.

The generation of large-volume image data currently is, and will, remain a constant for most plant phenomics experiments. There are two main considerations for analysis of image-based data. The first is access to image analysis tools to extract biologically meaningful digital traits. The second consideration is image storage, standardized cataloging protocols, and accessibility through public repositories so is that the data are widely available for the plant research community. Publically accessible tools for image analysis and image storage (e.g. Bisque/iPlant) have emerged in recent years, although this field still remains in its infancy (Kvilekval *et al.*, 2009; Yazdanbakhsh and Fisahn, 2009; Galkovskyi *et al.*, 2012; Tanabata *et al.*, 2012; Pound *et al.*, 2013; Brown *et al.*, 2014; Klukas *et al.*, 2014; Whan *et al.*, 2014; Fahlgren *et al.*, 2015; Müller-Linow *et al.*, 2015). However, this challenge is largely tractable because many of the image analysis resources that are publically available in the field of computer vision can be adapted for plant phenomics data. One such resource is OpenCV, which is a library of programming functions for processing many types of images (Bradski, 2000). However, adapting and utilizing these functionalities for plant phenomics requires computational and programming expertise, thus limiting wider utilization by the plant science community. This hurdle has been addressed with several timely resources, such as PlantCV, IAP, and others (Yazdanbakhsh and Fisahn, 2009; Galkovskyi *et al.*, 2012; Tanabata *et al.*, 2012; Pound *et al.*, 2013; Brown *et al.*, 2014; Klukas *et al.*, 2014; Whan *et al.*, 2014; Fahlgren *et al.*, 2015; Müller-Linow *et al.*, 2015).

Klukas *et al.* (2014) and Fahlgren *et al.* (2015) have both developed software for extracting biological information from images captured with LemnaTec platforms (http://www.lemnatec.com). Despite the power and flexibility provided by these programs, the loading and collection of experimental metadata from large datasets is done manually or through individual scripts developed by various research groups, making these tasks time-consuming and laborious. To put this in context, the number of files generated for a triplicated medium-scale experiment with 3 weeks of multi-camera imaging and ~300 genotypes can easily exceed three million. This can make even simple file management tasks computationally challenging for plant biologists. Moreover, the execution of image processing tasks is computationally intensive and requires access to high-throughput computational grids, and a proficiency in one or more programming languages. Many of the available plant image analysis software cannot easily be adapted by plant biologists for implementing processing workflows on computational grids, and require extensive modifications to fully realize the potential of distributing intensive computational tasks across an array of processors.

To address some of these challenges, we have developed Image Harvest (IH) as an open-source and flexible framework for making high-throughput image analysis accessible for plant biologists. Image Harvest (IH) is an open-source Python library for processing and analyzingplant images. In addition to providing powerful functions for processing several types of images (e.g. conventional color and fluorescence images), IH contains functions that greatly simplify the collection of metadata from the organizational structure of raw image databases. IH provides an option for implementing basic statistical functions and provides several definitions of digital traits to describe plant growth, morphology, and physiological responses. Moreover, IH has been integrated with the Open Science Grid (http://www.opensciencegrid.org/), which provides grid computing resources to academic users at no cost, thus reducing the overhead costs associated with the running and analyzing phenomics experiments. Here, we detail the image analysis functionalities of Image Harvest and demonstrate the value of some of the plant architecture-related digital traits by mapping them to the rice genome. The specific aims of this manuscript are: (1) to describe the creation and execution of processing workflows on a local machine and on the high-throughput computational cluster; (2) to demonstrate the processing power and accuracy of IH; (3) and to present some of the downstream applications of image-derived digital traits from two crop species.

## Materials and Methods

### Rice imaging at the late tillering stage

#### Plant materials

This study included 376 of the 421 original RDP1 rice (*Oryza sativa*) accessions (Famoso *et al.*, 2011; Zhao *et al.*, 2011; Eizenga *et al.*, 2014). Forty-five accessions were not included due to lack of seed availability or poor seed quality. Accessions were obtained from the USDA-ARS Dale Bumpers Rice Research Center and purified through single seed descent prior to phenotyping.

#### Plant Growth Conditions

Seeds from 373 genotypes from the rice diversity panel were surface-sterilized with fungicide (Thiram®) and germinated on moist paper towels in plastic boxes for 3 d (Famoso *et al.*, 2011; Zhao *et al.*, 2011). Three uniformly germinated seeds of each genotype were transplanted to pots (150mm diameter × 200mm height) filled with 2.6kg of UC Mix (Dimond, 1958).(Dimond, 1958). Square containers were placed underneath the pots to provide adequate water to saturate the soil. Plants were thinned to one seedling per pot six days after transplanting (DAT). For the first 20 DAT each pot was watered daily with ~150ml from the top of the pot. Over the course of the two experiments, the greenhouse temperatures during the day averaged 28.8 °C (±2.02 °C, SD) and 26.0 °C (±1.01 °C, SD) at night. Relative humidity was maintained at 63.4% (±9.04%, SD) during the day and 69.7% (±1.73%, SD) at night (Rotation Atomiser Defensor ABS3, Condair Ltd., Pfäffikon, Schwyz, Switzerland). At 21 DAT, each pot was watered to a uniform weight so that approximately 600ml of water was maintained in the soil. The experimental design was identical to that described by Campbell *et al.* (2015). Each pot was imaged daily using the LemnaTec Scanalyzer 3D system at the Plant Accelerator facility in Adelaide Australia for 18 days using two 5-megapixel RGB/VIS cameras (Basler Pilot piA2400-12gc) from three perspectives consisting of two side-view angles separated by 90° and a single top view. The entire data set of can be accessed through the iPlant Collaborative upon publication. (http://mirrors.iplantcollaborative.org/browse/iplant/home/shared/walia_rice_salt)

#### Phenotypic data analysis

Data were combined across experiments, and a linear model was fitted to calculate the adjusted means for each individual accession using the ‘lsmeans’ function in the LSMeans package in R (R Core Team, 2014; https://cran.r-project.org/web/packages/lsmeans/index.html). In the linear model, experiment is considered a fixed effect and accession as a random effect. The adjusted means were used for hierarchical clustering of raw moments using the complete-linkage method with Euclidian distance as the distance metric. For the comparison of 22 digital traits with three manual phenotypic measurements, Pearson correlation analysis was done using the rcorr function in the Hmisc package in R (https://cran.r-project.org/web/packages/Hmisc/index.html; R Core Team, 2014).

#### Genome-wide association mapping

All accessions were genotyped using 44 000 SNPs (Zhao *et al.*, 2011). A conventional mixed-model genome-wide association analysis was used to identify genomic regions associated with each of the 22 digital traits. The implemented mixed linear model can be summarized as: *y*=*Xβ*+*Cγ*+*Zu*+*e*, where *β* and *γ* represent coefficient vectors for SNP effects and subpopulation principal components, respectively, which are fixed effects, *u* is a random effect that accounts for population structure and relatedness, *Z* represents the corresponding design matrices, and *e* is the random error term. The model was implemented using EMMA in R using a minor allele frequency cut-off of 0.05 (Kang *et al.*, 2008; R Core Team, 2014).

## Results

### Software overview

Image Harvest (IH) is an open-source Python library for processing and analyzing plant images. IH provides integrated functions for complete image analysis from processing to descriptive statistics. The software is written in Python and uses algorithms from OpenCV to extract plant objects from complex images. SciPy is used for basic downstream statistical analysis of digital traits (http://www.scipy.org/index.html).

The implementation and execution of image processing steps varies depending on the nature and size of the input files. IH is available as stand-alone software for OS X, Windows, and Linux, and is also compatible for high-throughput image processing on computing clusters. The stand-alone software allows users to develop processing pipelines and process a small number of images. Once processing pipelines have been defined, they can be scaled up to process thousands of images in a high-throughput computing environment. IH workflows have been integrated into the Open Science Grid (OSG), which is comprised of ~100 000 opportunistically available processors (Pordes *et al.*, 2007). The presence of these workflows on OSG provides the high-throughput computing resources necessary for processing large image sets in a matter of hours for the plant science community. Although several software tools are available to perform image analysis, the ability to create distributed computing workflows is one of the salient and unique features of IH and is currently not implemented in other software.

#### Image capture

IH is a flexible platform, capable of processing images from various sources, ranging from automated imaging platforms to conventional hand-held cameras. The input images require the retention of straightforward parameters to ensure optimal processing of images and accuracy of digital traits. Imaging should be done in a uniform/controlled environment. It is recommended that plants be imaged in an environment with multiple light sources to minimize shadows and provide a uniform background for images (see Supplementary Fig. S1 at *JXB* online). The plant should be placed in front of a uniform background that is very different in color than that of the plant (for instance white, blue, or black). Based on our preliminary experiments, blue or white backgrounds are easiest to differentiate background features from plant pixels. These colors are also suitable for pots or frames that support the plant. If comparing multiple images, the camera and plant position should be fixed, and lighting conditions should be consistent from image to image. Slight movement caused by mechanical agitation or airflow may cause blurring, which reduces accuracy, and therefore the user should ensure that movement is minimized. Lastly, the imaging environment should be clear of plant matter, soil, or other debris, as these have similar colors properties to the plant and may be classified as ‘plant pixels’ during processing.

### Developing a processing workflow on a local computer

**Fig. 1.**
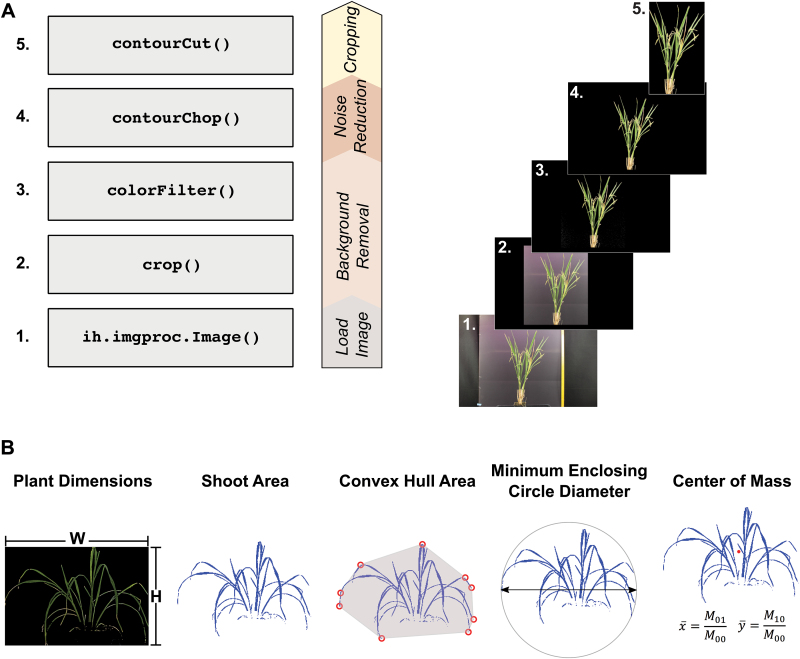
Simple processing workflow for side-view RGB images and digital morphological descriptors. (A) Major processing tasks used to process RGB side-view images of rice plants captured with a conventional SLR camera. The full processing script, which lists all processing tasks and the appropriate parameters is provided as Supplementary File S1.xls. The panels on the right show the results of each processing step. (B) A subset of the morphological digital traits currently available in IH. A complete list of the digital traits used is provided in Supplementary Table S3, and are visually depicted in Supplementary Fig. S3. The center of mass is indicated by the red point in the center of the plant.

Prior to implementing a computationally intensive workflow on thousands of images, an appropriate workflow should be developed on a local computer using a few representative plant images. IH allows for the results of each processing step to be monitored in real-time. Here we provide a simple processing pipeline for extracting plant pixels from a single color (RGB) image of a rice plant (cv 9311) at maturity (Fig. 1A). The images were captured using a conventional SLR camera (Canon Rebel T1i) and were imaged in a homemade imaging chamber to provide adequate lighting (see Supplementary Fig. S1). The raw images, as well as the full processing script is provided at the IH website (http://cropstressgenomics.org/data/html/ex_script_camera2.html).

Although individual workflows are specialized, there are common steps and design decisions in each workflow with wider applicability. For all image types, processing can be separated into four distinct modules: (1) background removal; (2) noise reduction; (3) cropping; and (4) data gathering. A list of functions available for each of the four classes of processing tasks is provided as Supplementary Table S1. The purpose of background removal is to remove a majority of the pixels that can clearly be defined as background, whereas noise reduction is fine-tuned for specific areas that may contain both background and plant pixels, such as the plant container. Finally, cropping is used to extract only the plant pixels from the image and, if performed effectively, increases data reliability.

#### Background removal

The first image processing step is a simple cropping function [crop()] to remove features outside of the background at the edges of the image. This step provides a more uniform background and allows the plant object to be extracted from the background using a few simple processing functions. In practice, the camera and plant positions will be fixed, so once parameters have been optimized for this function from a single image, the same can be applied to all other images. Next, the colorFilter()function is used to retain pixels with color qualities that satisfy a logical argument. Based on our logic, IH retains the pixels that meet the following condition: the green value of the pixel must exceed the blue channel value. colorFilter()can also be applied to a specific area of image by providing an optional region of interest (ROI) in the form [ystart, yend, xstart, xend]. These simple functions are sufficient to remove the majority of the background from the image.

#### Noise reduction and image cropping

The crop and colorFilter functions remove the majority of background pixels; however, other small background features remain in the image and must be removed through additional processing steps. These additional features are removed from the images based on their size: the contourChop() function is used to remove these small background features. The contourChop() function takes two arguments: the image to be processed and a minimum threshold area for contours to be removed.

Alternatively, IH has several morphological operations that can be used in place of contourChop() to remove small non-plant objects from the image. For example, morphological opening erodes all borders of the objects in the image and subsequently adds borders back to the shapes remaining after the erosion step. Thus, much of the noise in the background of the image can be removed.

The final step of image processing is to crop the image to just the plant to obtain an accurate measure of plant height and width. To do this, IH first generates a binary image and uses the contourCut() function to crop the image as a final step. The image is cropped so all contours that are greater than the specified area are included in the final output image.

### Executing processing workflows on a computing cluster

**Fig. 2.**
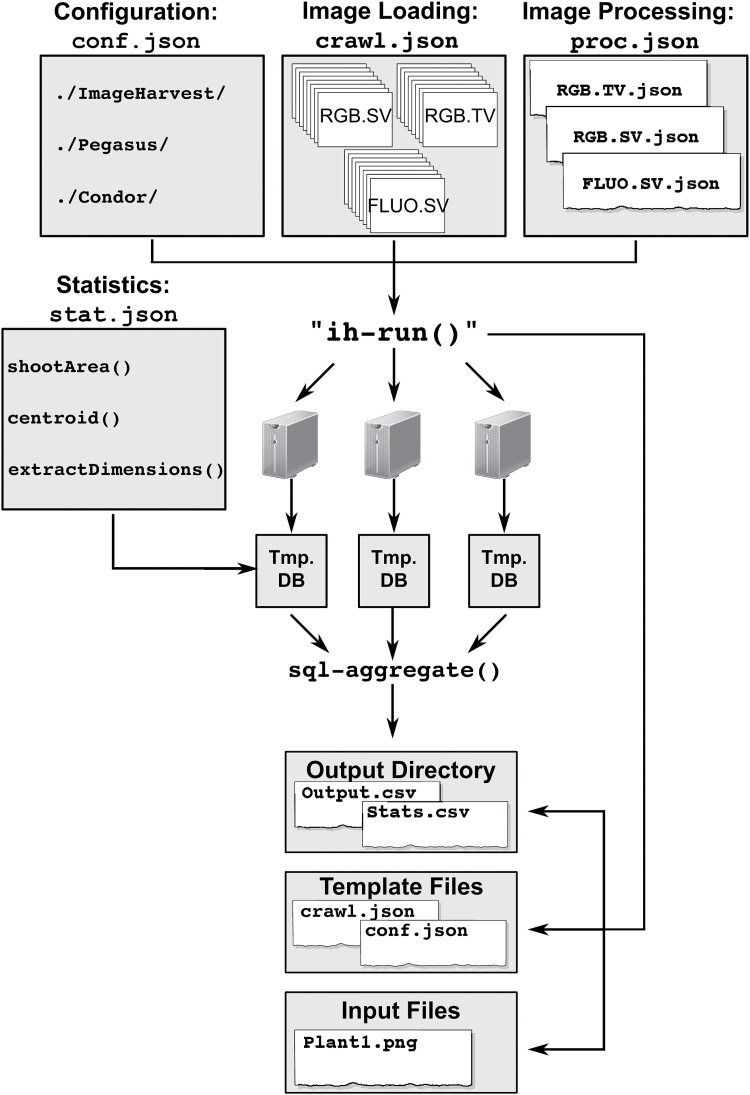
Schematic summarizing the creation and execution of supercomputing workflows using IH. IH requires four types of template files that contain the necessary information for image loading, processing, system configuration, and statistics workflows. The execution of the ‘ih-run’ command creates three directories, which contain the final output database, a copy of the submitted template files, and all non-image raw-input files. The ‘sql-aggregate’ function collects digital metric data from the intermediate databases, compiles the data and writes it to the output directory.

Once the user has established an appropriate processing workflow, the processing pipeline can be scaled to large, high-throughput data sets (>50 000 images) and executed on a high-throughput computing grid (Fig. 2). For datasets of this size, the execution of basic processing tasks is challenging. IH utilizes Pegasus for translating a series of computational tasks into a Directed Acyclic Graph and uses HTCondor to execute the jobs in parallel on a computing cluster (Thain *et al.*, 2005). Prior to initiating any workflow, the user should ensure that the proper environmental variables are established and Pegasus and HTCondor are installed on the computing grid. Alternatively, users may consider the OSG Connect service, which has IH and all the prerequisites installed, and is free to all academic users (Pordes *et al.*, 2007). Full-scale datasets are available at /stash/project/@RicePhenomics/public/. It is recommended that a structured directory be created that provides a detailed classification for each image (i.e. species, treatment, imaging date). The structure and organization of images are essential for the proper loading and creation of metadata.

The successful execution of workflows for processing large datasets relies on the creation of three JSON (JavaScript Object Notation) formatted template files: Image Loading, Image Processing, and Configuration. Below, a description and purpose for each template file is provided. The aim of this section is not to describe each processing step, as this has been discussed in the previous section, but to provide a brief overview of the necessary steps to execute workflow processing on computing clusters. An example of the necessary template files can be accessed through the IH website (http://cropstressgenomics.org/data/html/ex_workflow_1.html).

#### Image loading

For large image sets the loading and the creation of descriptors for each image can be a complex and laborious task. To circumvent these issues, IH uses an automated crawling process, which parses the information captured in the hierarchical structure and names of the directories to load metadata for images. The image loading step in the IH workflow takes care of two key problems posed by having large data sets, assuming the template file is written correctly. First, it tracks all images, and loads them into a single database, making it easy to locate and process files in the future. Second, all meta-data definitions are done as a result of the crawling.

The image loading template file will vary significantly based on how the images are named and organized. However, IH’s crawling function is designed to be flexible enough to be adapted to most directory structures. In this example, the images were captured with a LemnaTec Scanalyzer 3D and the pot identification number as well as the image time stamp are included in the directory names. Within each time-stamped folder there are several subdirectories that are named according to the image perspective (top view or side view) and image type (VIS/RBG or FLUO). It is assumed, if using a different system, that an appropriate method is in place to efficiently store and organize images after capture, as should be the case with other phenomics platforms that are capable of capturing thousands of images. Basic information such as the plant identification number, time-stamp information, and image type can be extracted from the directory naming and structure. Additional metadata, such as genotype names, replicate number, position in the greenhouse, etc. can be appended to the final results file by supplying a comma-separated file. The file should contain a key column, which matches the identifiers extracted from the directory names. Several examples of IH’s automated crawling are provided at the IH webpage (http://cropstressgenomics.org/data/). This allows the final results file to be navigated and mined with greater ease and simplifies downstream statistical analysis.

#### Processing

The image-processing template designates the type and order of image processing functions. Separate workflows should be designed for each image type (rgbsv, rgbtv, fluosv, etc.) and, as a result, the template may become quite long. Each workflow is defined as a list of jobs. The jobs are not necessarily executed in the order specified in the list, but the job definition should be structured such that it follows the list order as closely as possible for ease of reading. It is essential for the name of each workflow to match the name of the image types in the database.

#### Execution of IH and statistics workflows

The execution of complex workflows in IH is relatively easy provided the proper template files (Image Loading, Image Processing, and Configuration) and the directories have been established, and are fulfilled using the ih-run command. This creates a date and time-stamped directory, which contains the output database, a copy of the submitted templates and all non-image raw-input files. IH provides several functions for extracting digital traits that describe plant morphology, growth, and color/spectral qualities from processed images. A complete list of available functions is provided as Supplementary Table S1. These metrics can be combined from multiple views to describe different morphological traits.

**Fig. 3.**
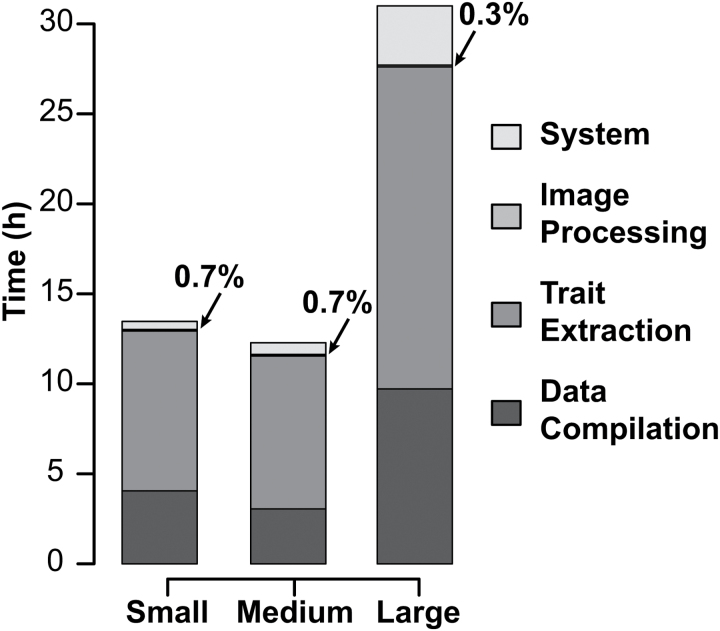
Computational time for image sets of various sizes. The processing speed of RGB and FLUO workflows was evaluated using three image sets of various size: small (19 475 images), medium (38 357 images) and large (77 665 images). The tasks were separated into four categories: system (Pegasus and condor related tasks), processing (actual image processing functions), trait extraction (‘ih-extract-multi’, ‘ih-stats-histogram’), and data compilation tasks (‘ih-sql-aggregate’). Time refers to the time experienced at the user’s end.

To demonstrate the performance of IH, we executed a processing workflow for FLUO side-view and VIS side- and top-view images using several image sets of varying size (77665, 38357, and 19457 images). Images were processed on a computing cluster (106 node LINUX cluster, Opteron 6272 2.1GHz, 4 CPU per node, 256 GB RAM per node). The small and medium image sets were completely processed in less than 13 hours, while the large image set consisting of >77K images were processed in slightly more than 30 hours (Fig. 3). The total time (from the user’s end) that is require to process image sets on a computing cluster is dependent on the number of tasks and the number of available processors. For the small and medium datasets (19457 and 38357 images, respectively), the number of available processors was probably greater than the number of tasks, which explains the nearly equal processing times. However, in the large dataset the number of available processors was far fewer than the number of tasks.

For all image sets the actual image-processing tasks accounted for less than 1% of the total processing times. Tasks involved with trait extraction and data compilation accounted for the majority of computation time. The function “ih-extract-multi” accounted for between 57–69% of the total computation time, while the “ih-sql-aggregate” function accounted for between 25–31% of total computation time. The “ih-extract-multi” is used to extract multiple metrics (color data, moment data, dimensions, etc.) that are chosen by the user from processed images. A full list of available metrics is provided as Supplementary Table S2. While the computation time for simple morphological traits is typically low, the color classification/histogram function implemented in this workflow requires considerably more time and is probably the major factor contributing to the large computation times for this task. IH uses Sqlite to manage processing outputs, which improves portability but requires a relatively time-intensive collation step. Although IH can extract digital metrics from multiple images, without running into concurrency issues, the data must be written to different databases at the worker nodes, which then must be aggregated into a single database. The “ih-sql-aggregate” function combines output from all individual databases to a single final database.

### Assessing plant growth with IH

**Fig. 4.**
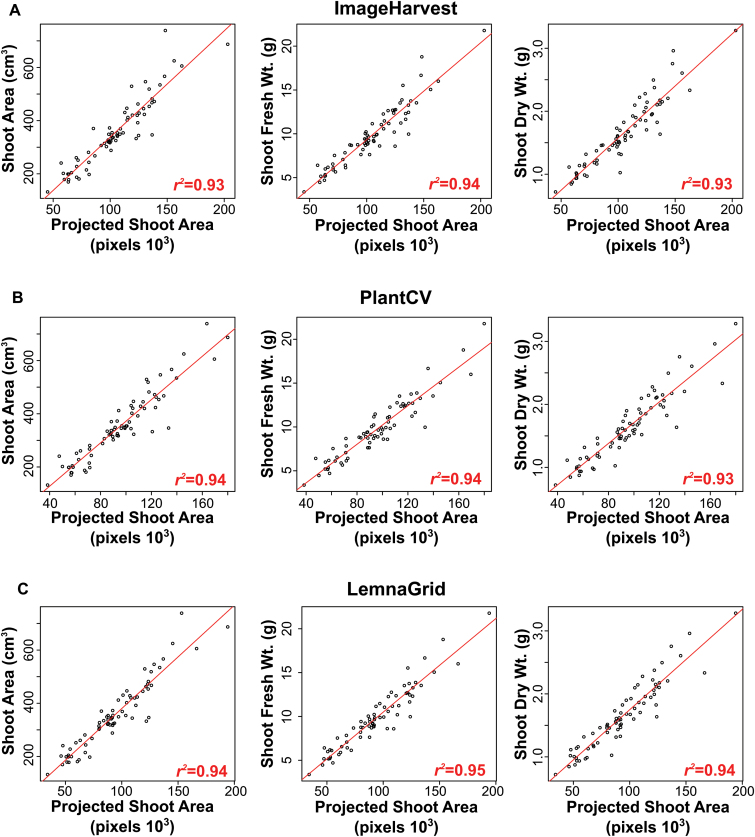
Comparisons between projected shoot area derived from several publically available plant image processing software programs and conventional phenotypic measurements of plant biomass. Projected shoot area, here defined as the summation of plant pixels from two side-view images, was calculated using (A) IH, (B) PlantCV, and (C) LemnaGrid and compared to three manual measurements of biomass. Manual phenotypic measurements were recorded on 72 rice plants 28 d after transplanting. Correlation analysis was performed using Pearson’s method. All correlations were significant (*P*<0.0001).

IH contains various functions for extracting digital traits describing plant shape, size, and color/spectral properties from the final plant object. A subset of these descriptors is illustrated in Fig. 1B, and the full list is available as Supplementary Table S1. Several software programs have been developed to process plant image data (e.g. Yazdanbakhsh and Fisahn, 2009; Galkovskyi *et al.*, 2012; Tanabata *et al.*, 2012; Pound *et al.*, 2013; Brown *et al.*, 2014; Klukas *et al.*, 2014; Whan *et al.*, 2014; Fahlgren *et al.*, 2015; Müller-Linow *et al.*, 2015). To compare the accuracy of IH with existing plant image analysis software, a dataset consisting of 144 RGB images of 72 rice plants during the early vegetative growth phase were processed using IH, LemnaGrid (www.LemnaTec.com), and PlantCV (Fahlgren *et al.*, 2015). Plants were imaged from two side-view angles and the foreground/plant pixels were summed from both side angles, and were used as a proxy for shoot biomass. This digital representation of shoot biomass, here termed projected shoot area (PSA), was compared to three manual phenotypic measurements (shoot area, shoot fresh weight, and shoot dry weight) recorded from the same group of plants. All software exhibited very high correlation with all phenotypic measurements (Fig. 4). Overall, the differences in accuracy among the three processing software products were minor. LemnaGrid displayed slightly higher accuracy than both IH and PlantCV. Both LemnaGrid and PlantCV displayed slightly higher correlation with shoot area (*r* ^2^=0.94) compared to IH (*r* ^2^=0.93). The results indicate that the accuracy of IH is comparable to other image processing software.

### From pixel-based digital traits to genes: a case study in rice

Selection for morphological traits, such as plant height, lodging resistance, and tillering capacity, are major contributing factors for increasing rice productivity over the past century. Rice breeders have defined specific ideotypes, which combine several traits to maximize photosynthetic capacity, growth habit, and grain production (Tandon and Jain, 2004; Peng *et al.*, 2008). However, classification and selection of ideotypes are highly subjective, and they are based on visual classification systems developed by plant breeders. The advent of image-based plant phenomics allows for the non-destructive evaluation of phenotypes while reducing human error, and provides an opportunity to define digital traits that describe multidimensional phenotypes and morphological features. In this section we aim to identify the major plant architectural classes from rice images, define digital traits that describe aspects of plant morphology, and demonstrate the potential to identify genomic regions associated with these digital traits. To this end, we imaged a panel of 376 diverse rice genotypes during the late tillering stage (41 d after transplant) using a RGB/VIS camera.

**Fig. 5.**
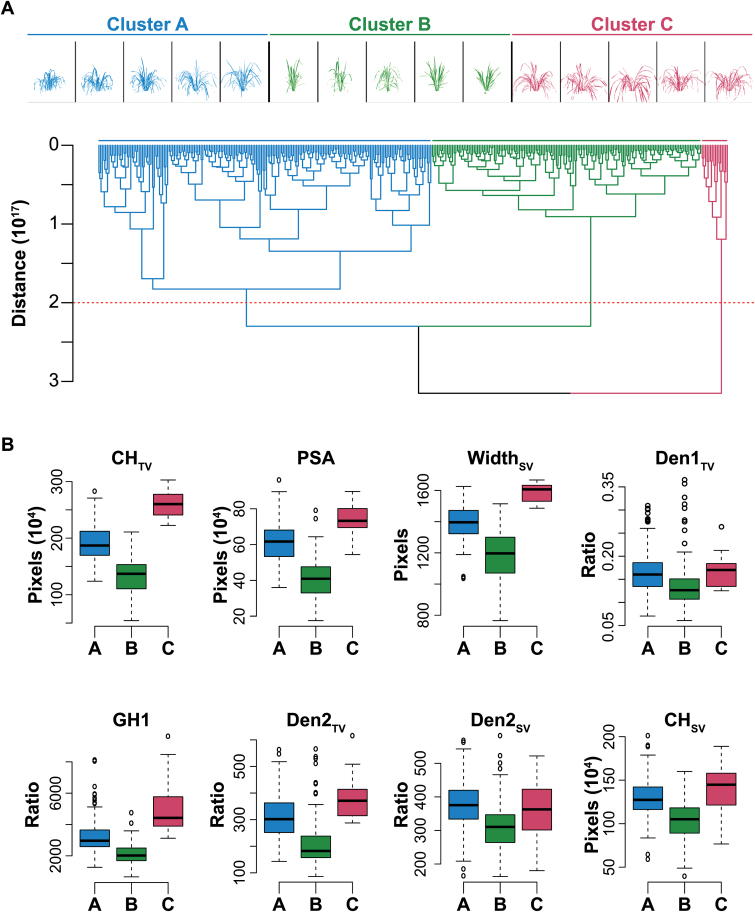
Hierarchical clustering of raw image moments (*M*) and the distribution of a subset of digital traits for each cluster. (A) Hierarchical clustering of raw image moments extracted from both side-view and top-view images of rice plants at 41 d after transplant. Five representative plants from each of the three clusters are shown above the dendrogram. A Euclidian distance threshold of 2×10^17^ was used to separate the clusters, and is indicated by the horizontal red dashed line. (B) Boxplots for eight of the 22 digital traits illustrating the phenotypic distributions of the major clusters. Boxplots for the remaining 14 digital traits is provided as Supplementary Fig. S2. Abbreviations: CH, convex hull; GH, growth habit; Den, density; PSA, projected shoot area; TV, top view; SV, side view.

To identify the major shape/plant architectural types in rice germplasm, raw image moments (*M*) were extracted from 1548 side-view (SV) and 774 top-view (TV) images and hierarchical clustering analysis was performed using the adjusted mean values for these digital metrics. Raw image moments extracted from binary images provide a numerical description of various properties of the foreground object, such as center of mass or orientation. Hierarchical clustering of raw image moments identified three major phenotypic groups (Fig. 5A). To determine how these groups differed in morphological features, a one-way ANOVA was performed using 23 digital traits that describe various morphological qualities (Fig. 5B, Suplemenatry Tables S3, S4). Significant differences between the groups (*P*<0.0001) were observed for 20 traits that describe plant width, compactness, and biomass (Fig. 5B, Supplementary Table S4). Interestingly, no significant differences were observed for traits corresponding to plant height, suggesting that these groups largely differ in biomass production and growth habit.

**Fig. 6.**
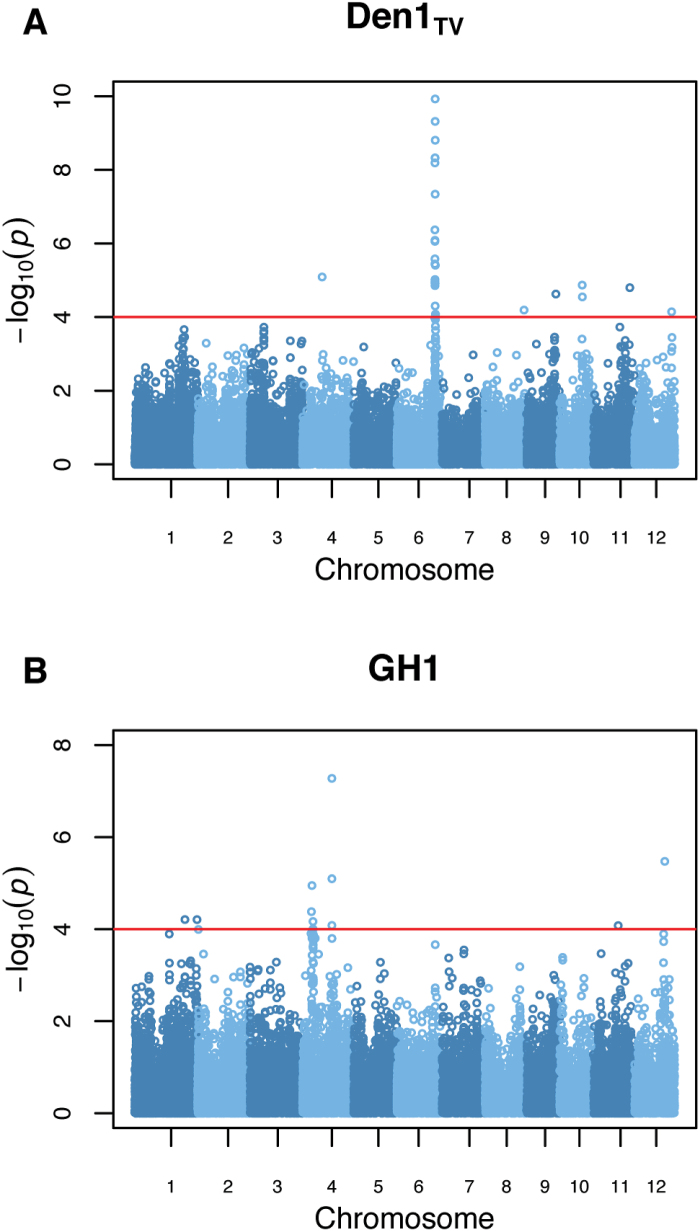
Genome-wide association analysis for Den1_TV_ (A) and GH1 (B). A threshold of *P*<10^−4^ was used to identify significant associations and is indicated by the red horizontal line in each plot. Abbreviations: Den, density; GH, growth habit; TV, top view; SV, side view.

To identify genomic regions associated with plant architecture, genome-wide association mapping was performed using 36 901 SNPs (single nucleotide polymorphisms) and the 23 digital traits for 359 genotypes (Supplementary Figs S2, S3). Three highly significant peaks were identified for Den1_TV_ and GH1, which may be proxies for canopy density and erect growth habit, respectively (Fig. 6). Notably, the most significant SNPs co-localize with genes previously associated with plant growth and development. A rice gene *OsRCN4* (LOC_Os04g33570) similar to *TERMINAL FLOWER1* (*TFL1*) in Arabidopsis was located approximately 25kb upstream of the most significant GWAS peak on chromosome 4 (see Supplementary Dataset S1). Moreover, several QTLs/genes known to regulate tillering, flowering time, and hormone homeostasis (Table 1) have been mapped to the long arm of rice chromosome 6, where a number of highly significant SNPs populated a region of ~861 Kb. The identification of significant associations with regions known to regulate plant morphology and flowering time suggests that digital trait outputs from IH can be a useful tool for the evaluation and selection of genotypes exhibiting optimal morphological and agronomic traits.

**Table 1.** Candidate genes and QTLs overlapping with other studies. GH, growth habit; Den, density; PSA, projected shoot area; CA, cropped area; Chr., chromosome

**Chr.**	**QTL position (bp**)	**Trait(s**)	**Candidate gene/ Reported QTLs**	**Reference**
4	20,144,020-20,622,471	GH1, GH2, GH3, GH4	*OsTFL1*	(Nakagawa *et al.*, 2002)
	13,268,374-13,668,374	Den1_TV_, Den3_TV_, Den2_SV_, PSA	*OsRERJ1*	(Kiribuchi *et al.*, 2004)
6	26,031,137-26,892,628	Den1_TV_, Den2_TV_, Den3_TV_, Den1_SV_, Den3_SV_, CA_SV_, Width_SV_	*qIN3-6*	(Yamamoto *et al.*, 2001)
			*qPH6-2*	(Duan *et al.*, 2013)
12	25,302,716-25,702,716	Den1_TV_, Den3_TV_	*OsIAA3*	(Nakamura *et al.*, 2006)

## Discussion

Plant phenomics is a new and powerful approach; however there are still considerable challenges for plant scientists to extract biologically meaningful information from images. Many existing algorithms have been developed in the field of computer vision and can be utilized to extract morphological and color properties from plant images. However, an extensive knowledge of several programming languages is necessary for the efficient implementation of these methods on large data sets, and may be a skill set that is rare in most plant biology research groups. To allow greater accessibility to these powerful algorithms in computer vision, IH combines utilities from OpenCV and SciPy, and provides a user-friendly, broadly applicable image-processing framework that can be run locally on conventional desktops to develop processing pipelines. Processing functions available in IH can be applied to images captured from several sources, such as handheld SLR cameras or phenomics platforms other than LemnaTec, and can be used to process various types of images (RGB and FLUO). In recent years several groups have released software to process images derived from phenomics platforms (e.g. Klukas *et al.*, 2014; Fahlgren *et al.*, 2015). IH has several advantages over existing software.

First, IH is an open-source software. While other proprietary software, such as LemnaGrid, work seamlessly with LemnaTec platforms, the user is required to purchase software licenses and is often confined to the processing algorithms and metrics offered within the software package. Although LemnaGrid offers numerous descriptive metrics, users often want to address biological processes beyond simple morphology, which may cause them to seek resources outside of LemnaGrid. For example, a binning strategy was used by Campbell *et al.* (2015) to identify color ranges in fluorescence images that were responsive to salinity treatment. At the time of publication, the methods that they implemented were not available in LemnaGrid. Other open-source software offers numerous metrics to describe plant morphology and color properties, and has also proved to be a means for new algorithms to be added, thus improving the flexibility of the software to address specific research needs (Klukas *et al.*, 2014; Fahlgren *et al.*, 2015).

Second, IH can process images generated from various sources, included other non-LemnaTec phenomics platforms, flatbed scanners, SLR cameras, and non-RGB images (fluorescence, hyperspectral, etc.). In addition to the pipelines presented above, several tutorials are provided at the IH website (http://cropstressgenomics.org/data/), which provide a complete description of pipelines for processing FLUO and RGB images, for phenomics platforms that use overhead cameras to phenotype trays of plants, and for quantifying morphological traits from images of seeds obtained with a conventional desktop scanner. Once a pipeline has been developed, the modular organization of IH functions then allows processing tasks to be easily translated to supercomputer workflows. In contrast, at the time of this publication, the functionalities of IAP are not easily accessible for images generated from platforms other than LemnaTec.

Third, IH has been developed to utilize the power of grid computing. Phenomics experiments produce a large amount of data, and although several user-friendly processing software options are available, the computational requirements for such datasets exceed the capacity of desktop computers or workstations. Access to linux-based computational clusters and a working knowledge of computer programing to integrate several existing software packages is necessary to process large image sets. IH circumvents these requirements by combining functionalities from OpenCV, SciPy, Condor, Sqlite, and Pegasus into a single toolkit that can be accessed by plant biologists with minimal programming expertise. Moreover, IH has been developed to be highly portable, thus allowing simple installation to most Linux-based computing grids with minimal configuration and assistance from system administrators.

The integration of IH with OSG provides the computational resources necessary for processing large image sets in a reasonable time. At the time of this publication, several software packages for processing images derived from phenomics platforms are publically available that have capabilities for parallelizing workflows. For example, PlantCV uses a terminal multiplexer, such as Tmux (https://tmux.github.io/) or Screen, to submit multiple processing jobs in parallel. However, these methods used for parallelization may not be adequate for realizing the full potential of super-computing facilities. Using this type of approach, computation is limited to the resources of a single machine. By contrast, with IH individual processing tasks within a workflow can be distributed to dozens or even hundreds of machines spread across the country in a manner that is transparent to the user, thus facilitating quick parallelization of processing tasks and analyses. This effectively allows any academic research group to develop and implement a processing workflow for large datasets without the need to purchase dedicated servers or workstations.

Image-based phenomics allows physiological and morphological traits to be quantified non-destructively at a high frequency throughout a given period. The images contain important digital traits that may provide phenotypes beyond what is typically quantified in conventional manual phenotyping. For instance, in the field of computer vision, image moments are used to describe various properties of a contour, such as the size or centroid of the object. Image moments have been used in plant and mammalian systems to classify or describe various features from images (Ortiz *et al.*, 2013; Rojo-Arreola *et al.*, 2014; Lin *et al.*, 2015). The applicability of IH functions for phenotyping large mapping populations are highlighted in the GWAS example presented above where 23 digital growth-related traits were quantified for 359 genotypes. The most highly significant signals were for metrics used to describe canopy density from top-view images (Den1_TV_, Den3_TV_) and GH1, which are traits that would be difficult to quantify manually for a population this size. Moreover, these SNPs were in close proximity to genes that are known to regulate plant morphology in rice or other species, indicating that these signals are biologically relevant. For instance, an *AtTFL1* ortholog was identified approximately 25 kB upstream of the most significant SNP associated with GH1, GH2, GH3, and GH4 on chromosome 4. While the role for this gene in rice is not known, *AtTFL1* represses the transition from vegetative to reproductive development in Arabidopsis (Shannon and Meeks-Wagner, 1991; Wickland and Hanzawa, 2015). In rice, ectopic overexpression of the *AtTFL1* orthologs *RCN1* and *RCN2*, prolong the vegetative growth phase and lead to an over-production of leaves (Nakagawa *et al.*, 2002). Although *OsRCN4* has yet to be characterized, the digital phenotypes exhibited by the allelic groups at the QTL on chromosome 4 are in agreement with the phenotypes exhibited by other *TFL1* homologs in rice.

In addition to *TFL1*, two genes were identified within QTLs that have been shown to regulate plant growth and morphology through hormones such as jasmonic acid (JA) and auxin. A QTL spanning ~400kb on chromosome 4 was associated with four digital traits (Den1_TV_, Den3_TV_, Den2_SV_, PSA). *OsRJR1* (*LOC_Os04g23550*) encodes a bHLH transcription factor and is located less than 2kb upstream of the most significant SNP for Den3_TV_ (id4003991). A study by Kiribuchi *et al.* (2004) showed that *OsRJR1* is a positive regulator of JA-mediated inhibition of shoot growth. While this study provided biological insight into *OsRJR1* function, further experimentation is required to elucidate the role of this gene in natural variation of plant morphology. A second gene known to be involved with auxin signaling was identified within a QTL associated with Den1_TV_ and Den3_TV_ on chromosome 12. *OsIAA3* is a member of the *Aux*/*IAA* transcription factor family, and has been shown to regulate auxin responses in rice (Nakamura *et al.*, 2006). Overexpression of *OsIAA3* resulted in auxin insensitivity, with transgenic plants exhibiting reduced crown root formation, shorter leaf sheaths, and abnormal leaf development, indicating that it is a negative regulator of auxin responses (Nakamura *et al.*, 2006). The identification of genes that are known to regulate morphology and development in rice and other species demonstrate that these digital traits may be associated with important biological processes.

Over the past decade the amount of genotypic data has increased substantially, which has allowed researchers to examine the genome and transcriptome at a high resolution and associate genomic data with a phenotype. However, phenotyping remains a significant bottleneck for bridging the genotype–phenotype gap. The advent of image-based phenomics allows large populations of plants to be phenotype non-destructively, and provides an opportunity to quantify phenotypes that were traditionally evaluated subjectively or those that are not visible with the human eye. Despite the large investment in the construction of phenomics facilities, few open-source software solutions have been developed that are capable of processing large datasets in a reasonable time. IH provides a framework to develop processing workflows and execute them in a distributed computing infrastructure.

## Availability and Requirements

**Project name:** Image Harvest

Project home page: http://cropstressgenomics.org/data/html/index.html; https://git.unl.edu/aknecht2/ih/

**Operating system(s):** Windows, Linux, OS X

**Programming language:** Python

**Other requirements:** Matplotlib 1.3.1+; NumPy 1.7+; OpenCV 2.4.x; GTK2 (including headers) 2.24+; blas 3.2.1+; lapack 3.2.1+; libav 0.5.3+; cmake 2.8.12+; ffmpeg 2.0.5+; Pandas 0.13.1+; Cython 0.21.1+; python-dateutil 1.4.1+; PyMeanShift 0.2.0+; SciPy 0.13.1+; Pegasus 4.4.0+; Java 1.8+; Perl 5.10+; HTCondor 8.3.0+

**License:** GNU GPL (https://git.unl.edu/aknecht2/ih/blob/master/GPL.txt)

**Any restrictions to use by non-academics:** *See GPL*

## Supplementary data

Supplementary data are available at *JXB* online. Full processing scripts and example images can be accessed at http://cropstressgenomics.org/data/.

**Figure S1.** Imaging environment used for phenotyping with a conventional SLR camera

**Figure S2.** Boxplots summarizing the phenotypic distribution within each cluster for 14 digital traits.

**Figure S3.** Visualization of 21 of the 22 digital traits derived from IH metrics.

**Table S1.** A complete list of functions that are available in Image Harvest.

**Table S2.** Arguments and metrics returned from “ih-extract-multi”.

**Table S3.** Digital traits used to describe plant morphological qualities.

**Table S4.** ANOVA results and phenotypic means for each digital trait and cluster.

**Dataset S1.** Candidate genes within a 200-kb window of significant SNPs: a window of 200kb was chosen based on the estimated linkage disequilibrium in rice (Zhao *et al.*, 2011).

Supplementary Data

## Abbreviations:

IH: Image Harvest
GWAS: genome-wide association mapping
OSG: Open Science Grid
QTL: quantitative trait loci
SNP: single nucleotide polymorphism
RGB: red, green, blue
FLUO: fluorescence
DAT: days after transplant
VIS: visible
GH: growth habit
Den: density
SV: side view
TV: top view
