# Supplementary material for: Image Harvest: an open-source platform for high-throughput plant image processing and analysis
Source: J Exp Bot. 2016 May 3;67(11):3587–99. doi: 10.1093/jxb/erw176 (PMC4892737; doi:10.1093/jxb/erw176)
Supplement: Supplementary Data [file supp_67_11_3587__index.html]

Image Harvest: an open-source platform for high-throughput plant image processing and analysis — Image Harvest: an open-source platform for high-throughput plant image processing and analysis — Supplementary Data 

# Image Harvest: an open-source platform for high-throughput plant image processing and analysis

## Supplementary Data

Data files

- supplementary\_dataset\_S1.xlsx - Supplementary Data
- supplementary\_figures\_S1\_S3\_tables\_S1\_S4.pdf - Supplementary Data
