# Supplementary material for: Image Harvest: an open-source platform for high-throughput plant image processing and analysis
Source: J Exp Bot. 2016 May 3;67(11):3587–99. doi: 10.1093/jxb/erw176 (PMC4892737; doi:10.1093/jxb/erw176)
Supplement: Supplementary Data [file supp_erw176_supplementary_figures_S1_S3_tables_S1_S4.pdf]

Image Harvest: An open source platform for high-throughput plant image processing and analysis

Avi C Knecht, Malachy T Campbell, Adam Caprez, David R Swanson, and Harkamal Walia

SUPPLEMENTARY DATA

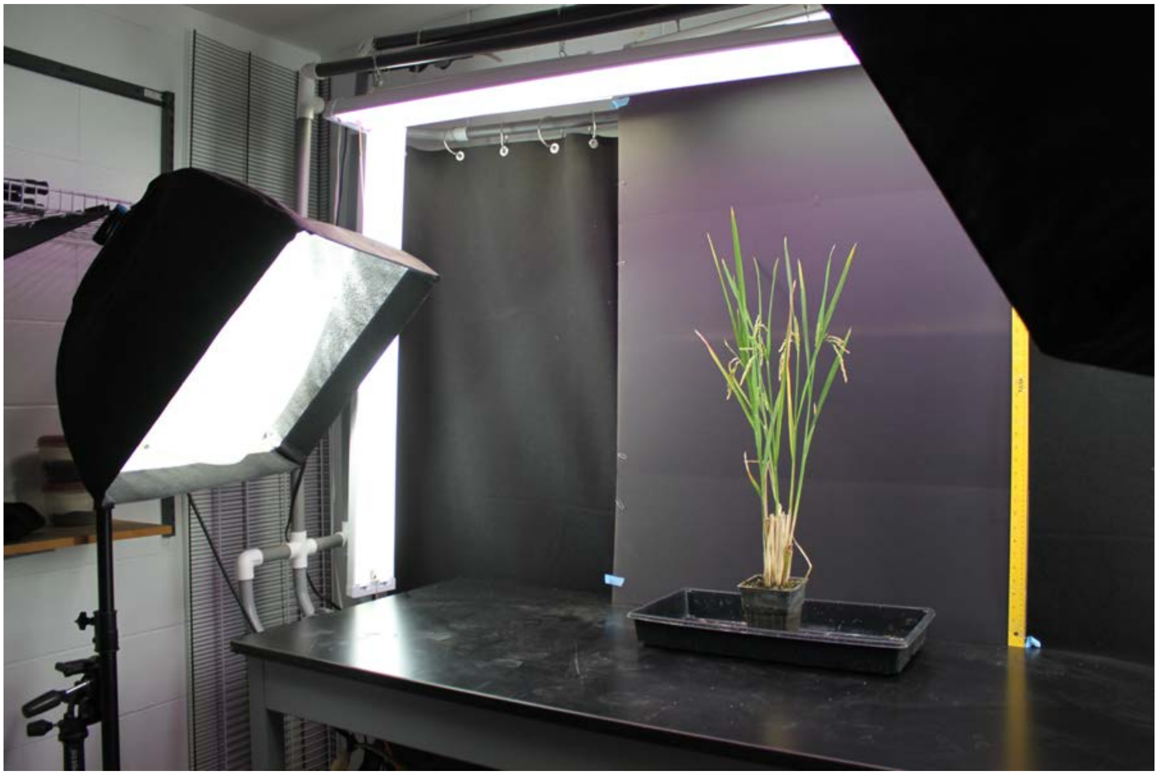

Figure S1. Imaging environment used for phenotyping with a conventional SLR camera. A imaging room was constructed to image a rice plant (cv 9311) at maturity in a homemade. To improve image processing several lighting sources were used to provide adequate light and a uniform background.

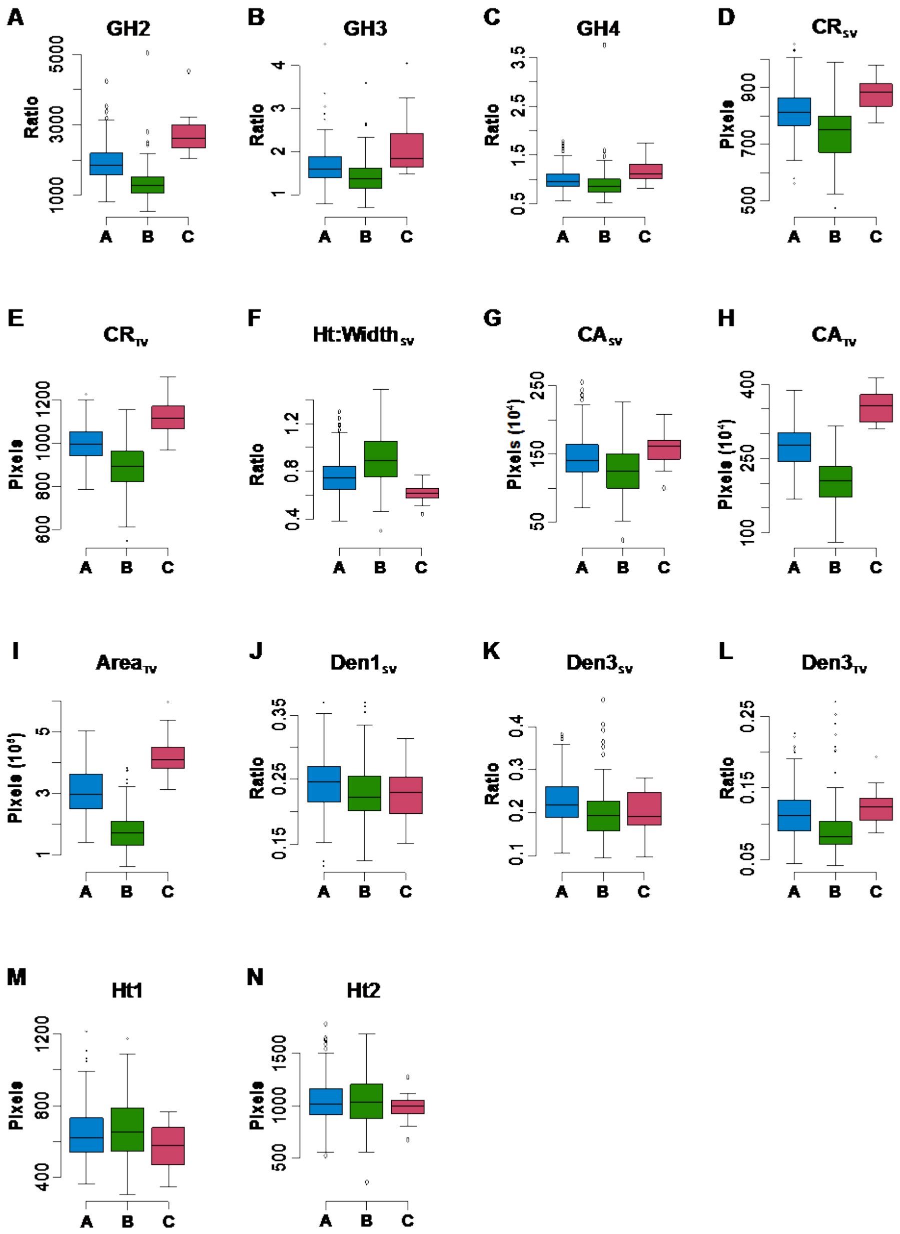

Figure S2. Boxplots summarizing the phenotypic distribution within each cluster for 14 digital traits (A-N). Hierarchical clustering of raw image moments identified three major phenotypic groups indicated by the letters below each bar. A full description of each of the digital traits, as well as the formula used for calculations is provided in Table S2. *Ht*: Height; *CH*: Convex Hull Area; *CR*: Radius of minimum enclosing circle; *Den*: Density; *GH*: Growth habit

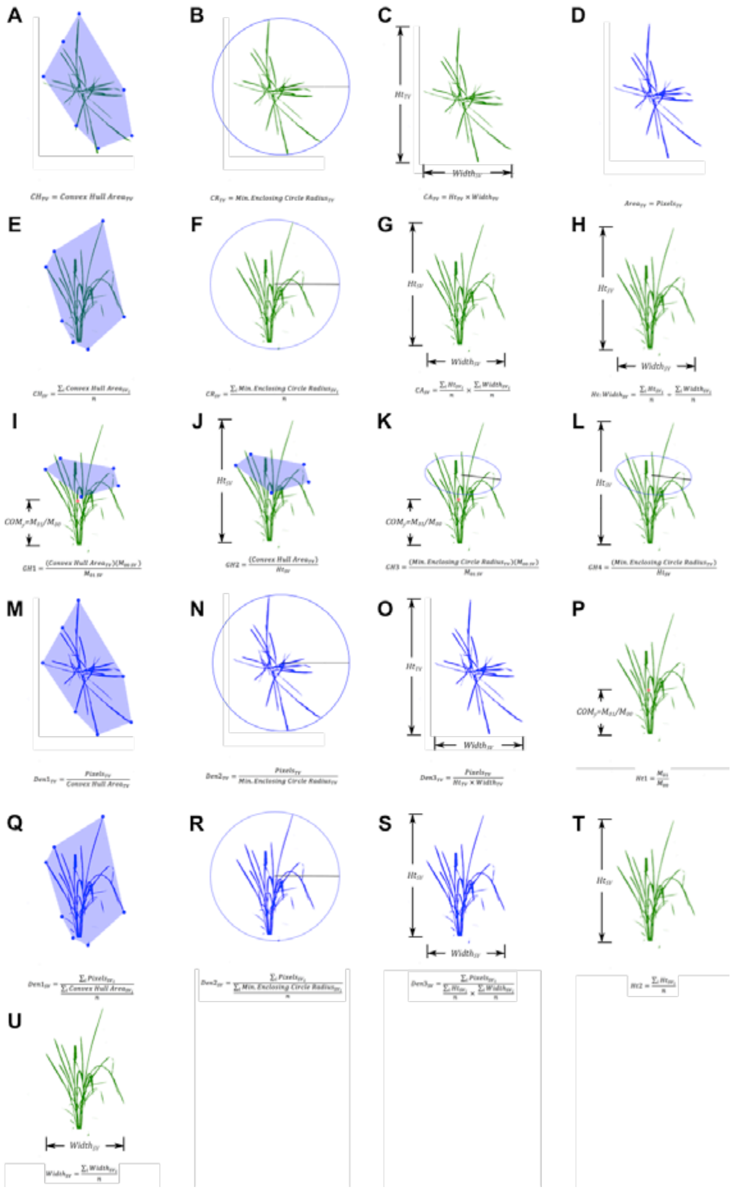

Figure S3. Visualization of 21 of the 22 digital traits derived from IH metrics (A-U). Each trait is visualized using a representative plant, and the equations used for trait calculations are listed below each panel. The summation of plant pixels from one or more imaging perspective was used in the calculation of several digital traits and is indicated by plant contours filled in blue. Convex hull area is indicated with the light blue shaded bounding polygon superimposed over portions of the plant. COM_y: Center of mass about the *y*-axis; *Ht*: Height; *CH*: Convex Hull Area; *CR*: Radius of minimum enclosing circle; *Den*: Density; *GH*: Growth Habit; *M*: raw moment; *n*: number of side view imaging perspectives.

Table S1. A complete list of functions that are available in Image Harvest.

Function	Function Class	Function Sub-class	Brief Description
destroy()	ih.imgproc.Image method	Base	Destroys all currently open windows.
list()	ih.imgproc.Image method	Base	Lists all saved states.
resize()	ih.imgproc.Image method	Base	Resize an image.
restore()	ih.imgproc.Image method	Base	Reloads a previously saved image from the ‘states’ variable.
save()	ih.imgproc.Image method	Base	This function saves the current image in the ‘states’ variable under the specified name. It can then be reloaded using the restore() method.
show()	ih.imgproc.Image method	Base	Displays the image in a window.
wait()	ih.imgproc.Image method	Base	Waits until a key is pressed, then destroys all windows and continues program execution.
write()	ih.imgproc.Image method	Base	Writes the current image to the given output directory, with the given name.
anova()	ih.statistics.Stats method	Data Analysis	Computes the analysis of variation of all numeric information based on 3 factors, treatment, date, and the interaction between the two. Analysis of variation is different than the rest of the stats functions, in that a lot of information is lost after running it. The results themselves correspond to columns (pixels, rmed, binx...) instead of actual images.
correlation()	ih.statistics.Stats method	Data Analysis	This function correlates all numeric values with values in the given file. The input data file is assumed to be in csv format.
logErrors()	ih.statistics.Stats method	Data Analysis	This function writes all errors from a given table to a log file, usually used at the end of image processing to write all images that did not process correctly.
export()	ih.statistics.Stats method	Data Analysis	This function simply extracts data from a database and writes it to csv format. Default functionality is to extract only data that has been processed.

Table S1 (con't). A complete list of functions that are available in Image Harvest.

Function	Function Class	Function Sub-class	Brief Description
extractPixels()	ih.imgproc.Image method	Post-process	Returns the number of non-black (foreground) pixels in the image. Creates a temporary binary image to do this.
normalize()	ih.statistics.Stats method	Data Analysis	Normalizes all numerical information to the specific column. This function is usually used with 'pixels' as the specified column, which expresses all numeric information as a percentage of the total pixels in the image.
shootArea()	ih.statistics.Stats method	Data Analysis	This function sums the numeric values of multiple image types together. In general, it is used to combine side view + top view images of the same spectrum.
treatmentComp()	ih.statistics.Stats method	Data Analysis	This function compares information between treatments – It finds plants that are identical except for treatment, and computes either a ratio or difference between them.
tTest()	ih.statistics.Stats method	Data Analysis	This function computes a ttest of the input table for all numeric headers.
extractBins()	ih.imgproc.Image method	Post-process	This function counts the number of pixels that fall into the range as specified by each bin.
extractColorData()	ih.imgproc.Image method	Post-process	This function calculates a normalized histogram of each individual color channel of the image, and returns the mean & median of the histograms for the channels specified.
extractConvexHull()	ih.imgproc.Image method	Post-process	Returns the area of the convex hull around all non black pixels in the image.
extractDimensions()	ih.imgproc.Image method	Post-process	Returns a list corresponding to the height and width of the image.
extractMinEnclosingCircle()	ih.imgproc.image method	Post-process	Returns the center and radius of the minimum enclosing circle of all non-black pixels in the image.
extractMoments()	ih.imgproc.Image method	Post-process	Calculates the moments of the image, and returns a dicitonary based on them. This function is a wrapper to the OpenCV function moments.
extractFinalPath()	ih.imgproc.Image method	Post-process	This function writes the absolute path of the output file to the database.

Table S1 (con't). A complete list of functions that are available in Image Harvest.

Function	Function Class	Function Sub-class	Brief Description
mask()	ih.imgproc.Image method	Processing	This function converts the image to a mask by performing convertColor("bgr", "gray"), convertColor("gray", "bgr")
adaptiveThreshold()	ih.imgproc.Image method	Processing	Thresholds an image by considering the image in several different windows instead of the image as a whole. This function is a wrapper to the OpenCV function adaptiveThreshold.
bitwise_and()	ih.imgproc.Image method	Processing	Performs logical AND between the input image and the comp image.
bitwise_not()	ih.imgproc.Image method	Processing	Inverts the image. If the given image has multiple channels (i.e. is a color image) each channel is processed independently.
bitwise_or()	ih.imgproc.Image method	Processing	Performs logical OR between the input image and the comp image.
bitwise_xor()	ih.imgproc.Image method	Processing	Performs exclusive logical OR between the input image and the comp image.
blur()	ih.imgproc.Image method	Processing	Smoothes an image using the normalized box filter. This function is a wrapper to the OpenCV function blur.
colorFilter()	ih.imgproc.Image method	Processing	This function applies a color filter defined by the input logic, to a targeted region defined by the input region of interest (ROI).
contourCut()	ih.imgproc.Image method	Processing	This function crops an image based on the size of detected contours in the image – clusters of pixels in the image. The image is cropped such that all contours that are greater than the specified area are included in the final output image.
crop()	ih.imgproc.Image method	Processing	This function crops the image based on the given region of interest (ROI) [ystart, yend, xstart, xend].
edges()	ih.imgproc.Image method	Processing	This function calculates the edges of an image using the Canny edge detection algorithm. This function is a wrapper to the OpenCV function Canny.
gaussianBlur()	ih.imgproc.Image method	Processing	This function blurs an image based on a Gaussian kernel.
convertColor()	ih.imgproc.Image method	Processing	Converts the given image between color spaces, based on the given types.

Table S1 (con't). A complete list of functions that are available in Image Harvest.

Function	Function Class	Function Sub-class	Brief Description
kmeans()	ih.imgproc.Image method	Processing	This function is a wrapper to the OpenCV function kmeans Reduced the number colors in the image to the most compact 'central' colors (k). The number of colors in the resulting image is the specified value 'k'.
knn()	ih.imgproc.Image method	Processing	Classifies and removes pixels based on the nearest neighbors algorithm. This function is a wrapper to the OpenCV function KNearest.
meanshift()	ih.imgproc.Image method	Processing	Segments the image into clusters based on nearest neighbors. This function is a wrapper to the pymeanshift module.
medianBlur()	ih.imgproc.Image method	Processing	This function smooths an image using the median filter.
morphology()	ih.imgproc.Image method	Processing	This function performs morphological operations based on the inputted values. This function is a wrapper to the OpenCv function morphologyEx.
normalizeByIntensity()	ih.imgproc.Image method	Processing	Normalizes each channel of the pixel by its intensity.
resizeSelf()	ih.imgproc.Image method	Processing	Resizes the current image.
threshold()	ih.imgproc.Image method	Processing	Thresholds the image based on the given type. The image must be grayscale to be thresholded.
dimFromROI()	ih.imgproc.Image method	Post-process	Returns a list corresponding to the height (defined as the distance from the upper most plant pixel to a upper boundary of a user-defined region of interest (ROI)) and width of the image.

Table S2. Arguments and metrics returned from “`ih-extract-multi`”.

Arguments	Description
--pixels	Counts the number of plant pixels in the image.
--colors	Calculates a normalized histogram of each individual color channel of the image, and returns the mean & median of the histograms for the channels specified.
--channels	This function extracts the total number of pixels of each color value (0 to 255) for each channel.
--moments	Calculates the moments of the image, and returns a dicitonary based on them. Spatial moments are prefixed with ‘m’, central moments are prefixed with ‘mu’, and central normalized moments are prefixed with ‘nu’. This function is a wrapper to the OpenCV function moments.
--hull	Calculates the area of the convex hull around all non black pixels in the image.
--circle	Calculates the center and radius of the minimum enclosing circle encompasing all non-black pixels in the image.
--dimensions	Calculates the height and width of the image.
--bins	Counts the number of pixels that fall into an RGB range. This function accepts a list of ranges, which is defined by six values (three minimum RGB and three maximum RGB values).

Table S3. Digital traits used to describe plant morphological qualities. SV: side view; TV: top view; *n*: number of side view images

Digital Trait	Class	Formula	Description
$Area_{TV}$	Biomass	$Area_{TV} = Pixels_{TV}$	Plant pixels from extracted from top view (TV) image
PSA	Biomass	$PSA = Pixels_{TV} + \sum_i Pixels_{SV_i}$	Summation of the plant pixels from all side view images and top view
$Ht{:}Width_{SV}$	Plant Shape	$Ht{:}Width_{SV} = \frac{\sum_i Ht_{SV_i}}{n} \div \frac{\sum_i Width_{SV_i}}{n}$	Ratio of the average height of the cropped SV image to the average width of the cropped SV image with dimFromROI()
CA_{TV}	Plant Size	$CA_{TV} = Ht_{TV} \times Width_{TV}$	Product of the height and width of the cropped TV image with dimFromROI()
$Den1_{SV}$	Density/ Compactness	$Den1_{SV} = \frac{\sum_i Pixels_{SV_i}}{\frac{\sum_i Convex\ Hull\ Area_{SV_i}}{n}}$	Ratio of the summation of the plant pixels from all SV images and the average convex hull area of all SV images
$Den2_{SV}$	Density/ Compactness	$Den2_{SV} = \frac{\sum_i Pixels_{SV_i}}{\frac{\sum_i Min.Enclosing\ Circle\ Radius_{SV_i}}{n}}$	Summation of the plant pixels from all SV images to the average radius of the minimum enclosing circle of all SV images
$Den3_{SV}$	Density/ Compactness	$Den3_{SV} = \frac{\sum_i Pixels_{SV_i}}{\frac{\sum_i Ht_{SV_i}}{n} \times \frac{\sum_i Width_{SV_i}}{n}}$	Summation of the plant pixels from all SV images to the average side view cropped area (CA_{SV}) with dimFromROI()
$Den1_{TV}$	Density/ Compactness	$Den1_{TV} = \frac{Pixels_{TV}}{Convex\ Hull\ Area_{TV}}$	Ratio of the plant pixels extracted from TV image to the convex hull area of the TV image
$Den2_{TV}$	Density/ Compactness	$Den2_{TV} = \frac{Pixels_{TV}}{Min.Enclosing\ Circle\ Radius_{TV}}$	Ratio of the plant pixels extracted from TV image to the radius of the minimum enclosing circle of the TV image
$Den3_{TV}$	Density/ Compactness	$Den3_{TV} = \frac{Pixels_{TV}}{Ht_{TV} \times Width_{TV}}$	Ratio of the plant pixels extracted from TV image to the top view cropped area (CA_{TV})
Ht2	Height	$Ht2 = \frac{\sum_i Ht_{SV_i}}{n}$	Average height of the final cropped image from all side views determined with dimFromROI()
Ht1	Height	$Ht1 = \frac{M_{01}}{M_{00}}$	Center of mass about the *y*-axis determined from the raw image moments (*M*) of side view images
GH1	Plant Shape	$GH1 = \frac{(Convex\ Hull\ Area_{TV})(M_{00\,SV})}{M_{01\,SV}}$	The ratio of the convex hull area of the TV image to the center of mass about the *y*-axis of all side view images
GH2	Plant Shape	$GH2 = \frac{(Convex\ Hull\ Area_{TV})}{Ht_{SV}}$	The ratio of the convex hull area of the TV image to the average height of all final cropped SV images determined with dimFromROI()

Table S3 (*cont'd*). Digital traits used to describe plant morphological qualities.

Digital Trait	Class	Formula	Description
GH3	Plant Shape	$GH3 = \frac{(Min. Enclosing\ Circle\ Radius_{TV})(M_{00\ SV})}{M_{01\ SV}}$	The ratio of the minimum enclosing circle radius of the TV image to the average center of mass about the y-axis of all SV images
GH4	Plant Shape	$GH4 = \frac{(Min. Enclosing\ Circle\ Radius_{TV})}{Ht_{SV}}$	The ratio of the minimum enclosing circle radius of the TV image to the average height all final cropped SV images determined with dimFromROI()
$Width_{SV}$	Plant Size	$Width_{SV} = \frac{\sum_i Width_{SV_i}}{n}$	The average width of all final cropped SV images
CA_{SV}	Plant Size	$CA_{SV} = \frac{\sum_i Ht_{SV_i}}{n} \times \frac{\sum_i Width_{SV_i}}{n}$	Product of the average height and width of all cropped SV images determined with dimFromROI()
CH_{TV}	Plant Size	$CH_{TV} = Convex\ Hull\ Area_{TV}$	Convex hull area of the TV image
CH_{SV}	Plant Size	$CH_{SV} = \frac{\sum_i Convex\ Hull\ Area_{SV_i}}{n}$	Average convex hull area of all SV images
CR_{TV}	Plant Size	$CR_{TV} = Min. Enclosing\ Circle\ Radius_{TV}$	The radius of the minimum enclosing circle radius of the TV image
CR_{SV}	Plant Size	$CR_{SV} = \frac{\sum_i Min. Enclosing\ Circle\ Radius_{SV_i}}{n}$	The average radius of the minimum enclosing circle radius of all SV images

Table S4. ANOVA results and phenotypic means for each digital trait and cluster. A one-way ANOVA was conducted to compare digital traits between each cluster. A threshold of $p < 0.0001$ was used to determine statistical significance.

Digital Trait	Cluster A		Cluster B		Cluster C		F-Value	p-Value
	Mean	SEM	Mean	SEM	Mean	SEM		
Ht2	1044.4	15.62	1053.6	18.97	984.4	34.50	0.684	0.5052
$Width_{SV}$	1391.3	7.63	1183.9	11.95	1586.2	14.60	157.799	2.2E-50
CA_{SV}	1456183.8	24100.20	1262268.2	28338.38	1564989.8	63311.77	16.467	1.40E-07
$Ht:Width_{SV}$	0.757	0.0117	0.904	0.0167	0.617	0.0191	37.579	1.35E-15
CA_{TV}	2757517.7	29851.35	2034003.6	37109.33	3544363.7	77959.15	169.499	4.33E-53
$Den1_{SV}$	0.242	0.0031	0.229	0.0037	0.225	0.0109	4.750	0.0092
$Den2_{SV}$	378.7	5.2217	313.6	5.5896	358.8	22.8188	35.224	9.72E-15
$Den3_{SV}$	0.222	0.0037	0.199	0.0047	0.203	0.0126	7.991	4.00E-04
$Area_{TV}$	306706.4	5372.40	178276.7	4972.82	425004.9	17803.17	195.642	7.88E-59
PSA	617370.6	7861.68	413742.7	8444.76	741479.0	23130.37	186.447	7.4E-57
$Den1_{TV}$	0.166	0.0033	0.137	0.0040	0.169	0.0090	16.637	1.20E-07
$Den2_{TV}$	312.7	6.08	204.5	6.49	385.1	21.00	89.241	2.13E-32
$Den3_{TV}$	0.116	0.0024	0.092	0.0030	0.124	0.0068	21.051	2.18E-09
Ht1	648.0	11.15	675.1	13.33	568.0	32.23	3.752	0.0244
CH_{TV}	1912150.1	22566.88	1343149.6	24169.27	2601697.5	61207.56	219.917	8.1E-64
CH_{SV}	1295565.6	16081.97	1039990.1	17641.92	1388537.5	72797.46	61.956	5.85E-24
GH1	3239.3	76.46	2101.9	51.31	5171.0	484.51	108.566	6.94E-38
GH2	1930.0	37.14	1356.9	38.07	2719.4	149.99	88.371	3.84E-32
CR_{TV}	992.2	5.81	887.0	9.04	1120.4	19.47	76.954	1.05E-28
CR_{SV}	820.5	5.47	747.5	7.63	879.6	13.91	41.195	6.84E-17
GH3	1.674	0.0341	1.409	0.0302	2.217	0.2116	30.140	7.35E-13
GH4	1.001	0.0157	0.905	0.0241	1.166	0.0541	10.822	2.70E-05
